# Unilateral Nerve Root Ligation for Multilevel Vertebral Column Resection After Fixed Post-infectious Deformity

**DOI:** 10.7759/cureus.9269

**Published:** 2020-07-19

**Authors:** Jeffrey Hatef, Justin Baum, John McGregor

**Affiliations:** 1 Neurosurgery, Ohio State University Wexner Medical Center, Columbus, USA

**Keywords:** kyphotic deformity, vertebral column resection, unilateral, nerve root preserving, spinal cord

## Abstract

Kyphotic deformity is a well-recognized complication of thoracic vertebral osteomyelitis, often requiring multi-level vertebral column resection for mobilization of the spine and reduction of the deformity. We present a case of severe post-infectious kyphosis treated with multi-level vertebral column resection via a unilateral approach. We obtained excellent decompression and deformity correction without neurologic decline. We review relevant literature regarding spinal cord blood supply and known potential complication of nerve root ligations.

## Introduction

Vertebral column resection (VCR) is a well-establish procedure for mobilizing severe, fixed spinal deformities in the thoracic spine [1]. The procedure involves accessing the anterior column of the spine via a costotransverse approach, traditionally requiring bilateral nerve root ligation. A unilateral approach has been described for the preservation of the contralateral nerve roots; however, this has been primarily limited to one-level vertebrectomy [2, 3]. Although bilateral ligation is generally well-tolerated at levels T2-T12, there is concern that bilateral nerve root ligations across multiple levels can result in ischemia to the spinal cord. We present a case about a 44-year-old patient who presented with severe fixed deformity after untreated osteomyelitis with a history of intravenous drug abuse. Near-complete resection of T7, T8, and T9 were accomplished through a unilateral costotransverse approach with preservation of the left-sided nerve roots and no neurologic worsening after surgery.

## Case presentation

**Figure 1:**
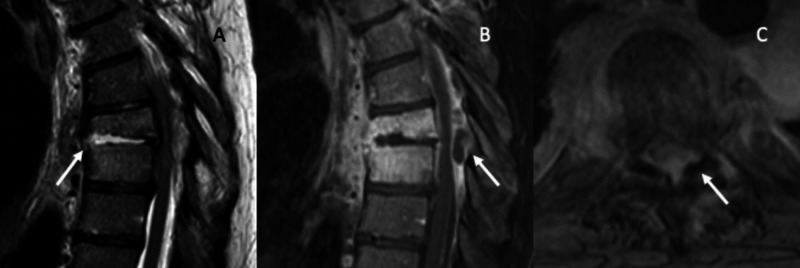
Initial imaging Preoperative imaging at initial presentation. A: sagittal T2 MRI showing discitis/osteomyelitis at T8/9 (arrow showing vertebral collapse). B: sagittal T1 with contrast MRI showing osteomyelitis/discitis at T8-9, arrow showing dorsal epidural abscess with severe spinal cord compression. C: axial T1 with contrast MRI demonstrating the severity of cord compression, arrow redemonstrating dorsal epidural abscess.

A 44-year-old female presented to our institution with three weeks of progressive back pain. On the morning of the presentation, she began to be unable to move her legs. She had two episodes of urinary incontinence. Her presenting physical exam showed no volitional movement in the lower extremities and no rectal tone, but preserved sensation to light touch and pinprick. MRI demonstrated osteomyelitis/discitis at T8 and T9 with a dorsal epidural abscess compressing the spinal cord (Figure 1).

She underwent emergency laminectomy and evacuation of the abscess. Operative and blood cultures grew methicillin-resistant Staphylococcus aureus; she was treated with intravenous vancomycin. After three weeks in the hospital, she was discharged to a skilled nursing facility. At her discharge, she was showing neurologic improvement and was able to flex her hips with 2/5 strength on the Medical Research Council (MRC) scale. She improved neurologically at the facility and left against medical advice.

**Figure 2:**
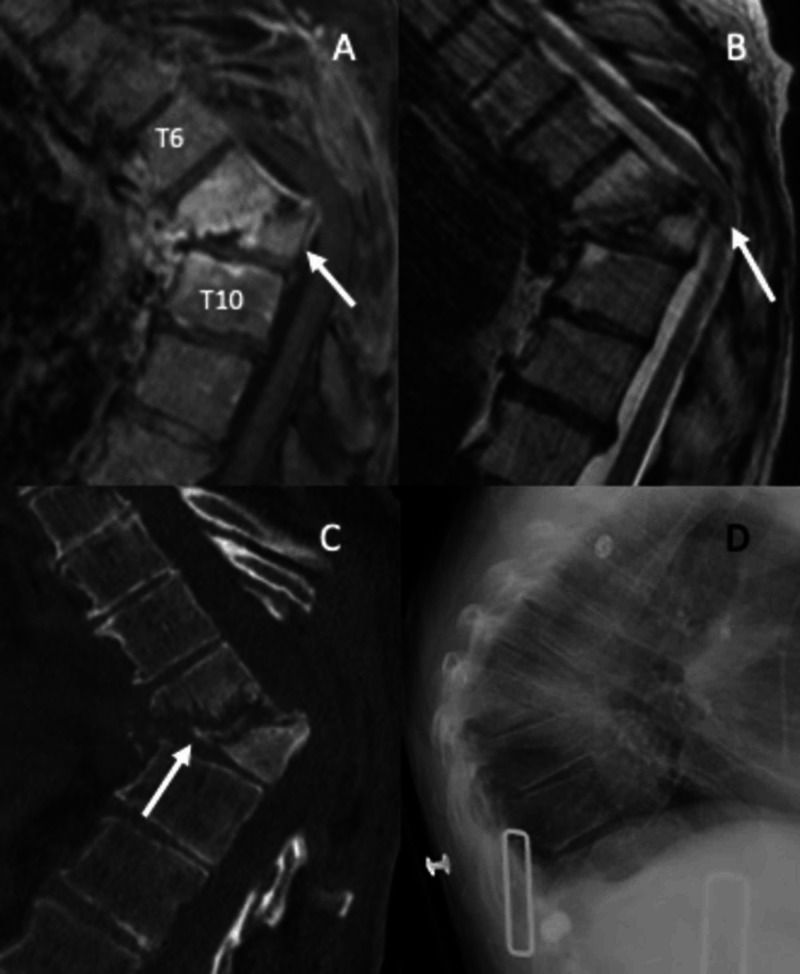
Pre-operative imaging A patient presenting several months after the initial epidural abscess evacuation with progressively worsening back pain and myelopathy. A: sagittal T2 MRI. B: sagittal T1 with contrast MRI. C: sagittal CT scan. D: sagittal XR upright in brace. Progression of osteomyelitis/discitis is evident with severe kyphotic deformity and destruction of the T7, T8, and T9 vertebral bodies. Arrows: A/B - severe kyphotic deformity showing the severity of spinal cord compression; C - erosive changes within multiple vertebrae consistent with osteomyelitis.

She again presented to our institution several months later with severe back pain and slowly progressive inability to ambulate. She had brisk reflexes in the lower extremities, sustained clonus in both feet, and sensory changes below a T7 dermatome. Her motor examination revealed MRC 3/5 weakness in bilateral hip and knee flexion and 2/5 weakness in bilateral dorsi- and plantarflexion. Imaging at that time revealed a significant post-infectious deformity (Figure 2).

There was the destruction of T7-9 with angular focal kyphosis measuring 55 degrees. CT scan showed evidence of fusion among the involved vertebral bodies. After a brief course of pre-operative antibiotics, she underwent deformity correction and spinal cord decompression.

The patient underwent instrumented posterior spinal fusion from T2-L1 with VCR at T7 - T9. Intraoperative neuromonitoring of somatosensory evoked potential (SSEP), motor evoked potential (MEP), and electromyography (EMG) were used throughout the case. Signals from SSEP were symmetrically decreased, but there were no monitorable signals in the lower extremities in MEP. The spine was exposed subperiosteally from T2-L1, and pedicle screws were inserted bilaterally from T2-T6 and T10-L1. The facet joints at the level of the prior laminectomy were found to be fused. Fluoroscopy was used to identify the level of the osteotomy; access to the vertebral bodies was limited by the sixth to ninth ribs. Bilaterally, ribs six, seven, eight, and nine were exposed for approximately four centimeters, dissected circumferentially, and resected. On the left, the facet joints were removed, and the remaining pedicles were flushed to the vertebral bodies. On the right, the T7, T8, and T9 nerve roots were doubly ligated and divided, and the pleura was dissected from the vertebra using monopolar electrocautery until the anterior aspect of the spine was identified.

A temporary rod was placed on the left spanning T5 - T11 before anterior resection began. The T6/7 and T9/10 disc were identified with fluoroscopy and incised to outline the extent of the osteotomy. The vertebral were decorticated with a burr and de-cancellated using osteotomes and curets. There were no identifiable disc spaces, and the deformed vertebra were solidly fused. After the majority of the bodies were removed, the compressive fragments in the canal were gently tamped into the cavity.

**Figure 3:**
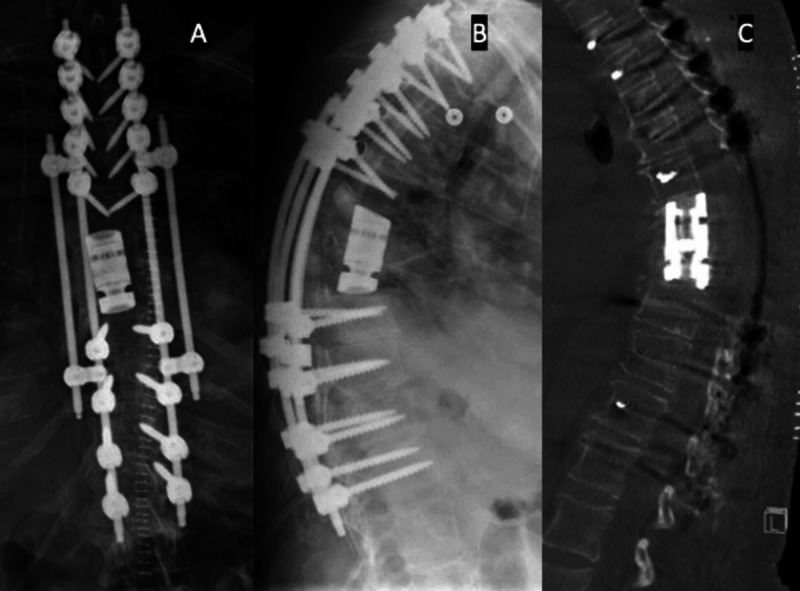
Post-operative imaging Postoperative imaging after the patient underwent vertebral column resection for the treatment of severe kyphotic deformity. A: anteroposterior X-ray. B: lateral X-ray showing T2-L1 instrumentation and intervertebral cage placement. C: sagittal CT scan reveals a significant reduction of kyphotic deformity.

We worked from the right until the contralateral pedicles were palpated and visualized. We then turned our attention to completing the osteotomy through the left side. We retraced the T6 nerve root and used a burr to remove the lateral vertebral body wall abutting the T6/7 disc space. We similarly completed our osteotomy through the T9/10 disc space. By resecting over 90% of the vertebra and leaving only a thin shell anteriorly and laterally, we adequately released the deformity. To correct the kyphosis, we first placed a second temporary rod on the right side. We used in-situ bending to induce lordosis in the spine, opening the anterior column. We placed an appropriately sized expandable cage and compressed posteriorly to further reduce kyphosis. Spinal fusion was performed with allograft and autograft. The patient awoke at her neurologic baseline and discharged on post-operative day ten. Post-operative CT and X-rays are seen in Figure 3.

She has seen improvement in her neurologic function to the American Spinal Injury Association (ASIA) score D and can ambulate short distances with assistance. There has been no loss of correction or instrumentation failure at a one-year follow-up.

## Discussion

Our patient presented with an unusually severe post-infectious deformity with severe compression of the spinal cord and progressive myelopathy. Deformity correction and neural decompression are required to maximize the clinical outcome. Traditionally, anterior decompression and fusion through a trans-thoracic approach would offer excellent exposure without neural compression or sacrifice. However, our patient presented with evidence of anterior and posterior fusion, which would severely limit the amount of deformity correction that could be obtained anteriorly. Staged anterior and posterior decompression and release procedures would allow correction but at the expense of multiple morbid procedures. Vertebral column resection allows for a single-stage decompression and deformity correction via a posterior approach and was selected as the most appropriate option in this case [4].

As first described, VCR requires bilateral nerve root sacrifice to provide access to the anterior column of the spine for decompression, mobilization and grafting. Nerve root sacrifice at one or two levels is a common neurosurgical procedure that is generally well tolerated. Multilevel resections for spinal tumors with nerve root ligation has been shown to be safe [5]. However, as the number of roots sacrificed increases, the risk for spinal cord ischemia increases; delayed and immediate neurologic deficits have been described after nerve root and segmental artery ligation during spinal fusion [6, 7]. Additionally, paraplegia has been described after ligation of multiple nerve roots during chest wall resections involving the costovertebral joints [8]. Although a unilateral approach to thoracic decompression and cage placement has been described, it has been limited to one- and two-level transpedicular decompressions [2, 3]. In our case, we were able to resect 90% of the involved vertebrae across three levels and significantly reduce kyphosis.

The primary blood supply to the spinal cord is through the anterior and posterior spinal arteries, which generally provide a longitudinal blood supply to the cord. The traditional teaching is of a dominant supply through the artery of Adamkiewicz, located anywhere between T8 and L3 on the left, with 50% of arteries at T9 or T10 [9]. However, clinical evidence has demonstrated that as the number of nerve roots or segmental arteries ligated, as opposed to simply ligation of the artery of Adamkiewicz, poses a higher risk of vascular injury to the spinal cord [10, 11]. This has been seen in anterior and posterior spinal fusions and thoracoabdominal aortic aneurysm repairs. The blood supply to the cord is tenuous at this level; although rare, spinal cord infarction is a well-described and disastrous potential complication of multiple nerve roots ligations at this level.

In our case, we made the decision to go forward with an all-posterior approach. As the patient already had severely compromised spinal cord function, we felt it was prudent to take additional measures if multiple nerve root ligations are required. One option is to place a temporary aneurysm clip across the roots in question. SSEP and MEP signals are monitoring for 5-10 minutes, and if no changes are seen in spinal cord function, the roots can be safely ligated [12]. However, SSEP alone has not been a reliable marker of motor function during spinal surgery [13, 14]. Neuromonitoring did not show monitorable MEP, so this was not an option for our patient. We made the decision to ligate roots only on the right side and use a unilateral approach for bilateral vertebral resection. Although a coronal deformity may influence the approach side, our patient had a symmetric deformity. We chose the right side to specifically preserve those roots that may be involved in the blood supply to the cord arising from the aorta on the left. We were able to completely decompress the cord and mobilize the spine above and below the deformity with the vascular compromise to the spinal cord.

Our report does have limitations to further generalization. Although we preserved the left-sided nerve roots, this came at the expense of significant rib resection to allow for adequate visualization ventral to the cord. Although our patient did not suffer a significant pulmonary complication, the number and extent of ribs removed can certainly contribute to respiratory insufficiency. In this case, we treated a symmetric deformity; it is likely that a significant coronal deformity would not be sufficiently mobilized without a bilateral approach. Although there was no neurologic decline after surgery, we cannot be sure that is due to the unilateral root sacrifice. It is possible that the involved roots were not important for spinal cord supply, or perhaps the slow degree of deformation and compression allowed for collateral supply to form. Despite these limitations, we believe that it is sensible to avoid multilevel bilateral root sacrifices. We were able to accomplish all surgical goals without exposing the patient to undue risk.

## Conclusions

VCR is a complex procedure reserved for the most severe thoracic spinal deformities. Although traditional bilateral approaches are safe for one- and two-level resections, larger multilevel VCR with bilateral nerve root ligation have a risk of spinal cord ischemia, especially in patients with high grade compression and myelopathy. We show that in a patient with severe, fixed post-infectious deformity, unilateral nerve root ligations allowed excellent ventral exposure for decompression, along with adequate release for deformity correction. Further studies are needed to delineate the precise role for unilateral approach to VCR.
